# Impetus to change: a multi-site qualitative exploration of the national audit of dementia

**DOI:** 10.1186/s13012-020-01004-z

**Published:** 2020-06-17

**Authors:** Michael Sykes, Richard Thomson, Niina Kolehmainen, Louise Allan, Tracy Finch

**Affiliations:** 1 Newcastle University, Newcastle upon Tyne, UK; 2 University of Exeter, Exeter, UK; 3 Northumbria University, Newcastle upon Tyne, UK

**Keywords:** Audit and feedback, Dementia, Quality improvement, Assurance, Performance measurement, Qualitative research

## Abstract

**Background:**

National audit is a key strategy used to improve care for patients with dementia. Audit and feedback has been shown to be effective, but with variation in how much it improves care. Both evidence and theory identify active ingredients associated with effectiveness of audit and feedback. It is unclear to what extent national audit is consistent with evidence- and theory-based audit and feedback best practice.

**Methods:**

We explored how the national audit of dementia is undertaken in order to identify opportunities to enhance its impact upon the improvement of care for people with dementia. We undertook a multi-method qualitative exploration of the national audit of dementia at six hospitals within four diverse English National Health Service organisations. Inductive framework analysis of 32 semi-structured interviews, documentary analysis (*n* = 39) and 44 h of observations (*n* = 36) was undertaken. Findings were presented iteratively to a stakeholder group until a stable description of the audit and feedback process was produced.

**Results:**

Each organisation invested considerable resources in the audit. The audit results were dependent upon the interpretation by case note reviewers who extracted the data. The national report was read by a small number of people in each organisation, who translated it into an internal report and action plan. The internal report was presented at specialty- and organisation-level committees. The internal report did not include information that was important to how committee members collectively decided whether and how to improve performance. Participants reported that the national audit findings may not reach clinicians who were not part of the specialty or organisation-level committees.

**Conclusions:**

There is considerable organisational commitment to the national audit of dementia. We describe potential evidence- and theory-informed enhancements to the enactment of the audit to improve the local response to performance feedback in the national audit. The enhancements relate to the content and delivery of the feedback from the national audit provider, support for the clinicians leading the organisational response to the feedback, and the feedback provided within the organisation.

Contributions to the literature

- Audit and feedback is a much used intervention to improve care. For example, over 98% of English and Welsh hospitals take part in the national audit of dementia.
- Evidence and theory describe components of audit and feedback associated with greater improvement.
- We describe organisation-level decision-making and a lack of clinician-level feedback as part of the national audit of dementia, and propose opportunities to improve impact.
- These findings contribute to work to enhance national audit and highlight the importance of organisation-level processes to the effectiveness of audit and feedback.

## Background

More than one in four people in acute hospitals have dementia [1]. People with dementia do not always receive identified best care; for example their pain may not be assessed, and carers may not be asked about causes of distress and what might calm the patient if they become agitated [1]. The national audit of dementia is a mandated [2] intervention to improve the care for people affected by dementia. The national audit of dementia provides feedback to National Health Service (NHS) organisations about the quality of care received by people with dementia in general hospitals. The audit is commissioned on behalf of NHS England and has taken place approximately every 2 years since 2010.

Audit and feedback is effective, but there is variation in the extent to which it improves care. A Cochrane review of audit and feedback [3] found that a quarter of audits stimulated less than 0.5% improvement, but a quarter exceeded 16% improvement. A low baseline was associated with increased improvement, as was the use of feedback that was repeated, given by a supervisor or peer, given in writing and verbally, and that included specific targets and an action plan [3]. Further factors that may be associated with increased effectiveness of audit and feedback draw upon theory: Colquhoun et al. [4] identified hypotheses to enhance audit, describing the potential roles related to the feedback recipient (e.g. the trust they have in the data), the target behaviour (e.g. the extent to which barriers to improvement can be addressed), the content of the audit and feedback (e.g. whether benchmark comparisons are included) and the delivery of the audit and feedback (e.g. if it presented at the time of decision-making about care). More recently, Brown et al. [5] synthesised evidence and theory about audit and feedback to develop clinical performance feedback intervention theory (CP-FIT). Briefly, CP-FIT describes factors associated with the feedback, the recipient and the context which act through diverse mechanisms (e.g. social influence, actionability) upon how the feedback is perceived, and whether intention and behaviour to improve clinical performance is produced. There have been calls to test the impact on care of the implementation of potential evidence- and theory-based enhancements to audit and feedback [6].

In order to identify potential enhancements to an existing audit, it is necessary to understand current audit and feedback practice. Specifically, we aimed to describe the content and delivery of the national audit of dementia and identify potential enhancements.

## Methods

This multi-method study used interviews, observations and documentary analysis, supported by stakeholder involvement through two groups: a co-production and an advisory group. Multi-methods enable the identification of reported practices and influences, as well as of tacit knowledge and practices taken for granted [7, 8].

### Setting

We studied six hospitals (approximately 4750 beds) within four English NHS organisations, purposively sampled to maximise diversity. We identified organisations with diverse regulator (Care Quality Commission) ratings for clinical effectiveness, sought hospitals within each rating that were of different sizes (full-time equivalent staff ranged across organisations from 4000 to 15000) and reviewed their previous performance on the national audit (Table 1). Consideration of both hospital and organisation level was important as national audit of dementia feedback is provided at the hospital level, but staff are employed at the organisation-level. Some hospitals at some sites did not meet the inclusion criteria of the national audit. All hospitals receiving national audit of dementia feedback at each site were included in the study. The sample of interviews, observations and documents (Appendix 1) was informed by co-production group input (described below) and emerging findings of the study. The sample sought people, events and documents able to provide diverse perspectives upon the content and delivery the national audit.

**Table 1:** A description of the sites and sample

Site (organisation)	Hospitals in study	Regulator assessment (rated 2014 to 2016)	Interviews	Observations	Documents
A	2	Requires improvement	9	18	14
B	1	Good	8	10	7
C	2	Outstanding	10	8	14
D	1	Requires improvement	5	0	4

### Interviews

MS interviewed 32 purposively sampled staff, undertook 36 observations (44 h) and analysed 39 documents across the sites. Interview participants (Appendix 1) were accessed through the hospital research department, approached by email and gave written informed consent, as described below. Interviews were semi-structured, conducted face-to-face and audio-recorded. The topic guide (Appendix 3) explored participants’ involvement with audit, their reported perception of why it was undertaken, what happens during audit and feedback, what affects its effectiveness and what could be changed to increase effectiveness. There were also questions targeted based upon earlier findings and the participants’ role. Mean interview length was 59 min (range 36 to 98 min). Whilst the period of interviews overlapped with the period of observations and documentary analysis, they happened during separate site visits. Concurrent data collection enabled findings from different sources to inform sampling and data collection (e.g. interview responses and documentary analysis targeting later observations, observations informing the interview topic guide). During the first three interviews, MS drew a diagram representing that participant’s description of what happens during audit (See example in Appendix 2). The diagram was shared with the respective participant for amendment during the interview. The diagrams were discussed with the co-production group and collated into a single diagram. This collated diagram (Appendix 2) was used (and further developed) in later interviews as part of an amended interview topic guide.

### Observations

Observations involved 204 staff participants at ward, specialty- (e.g. dementia steering group, care of the older person governance group) and organisation-level (e.g. organisational quality committee), meetings to plan, prepare for and respond to the national audit and the gathering and recording of national audit data. Participants were accessed through the hospital research department, approached by email and gave informed consent, as described below.

Observations took place at three of the four organisations, with the researcher taking field notes and asking exploratory questions [9]. Mean observation length was 74 min (range 14 to 226 min). Reflective notes were taken after both interviews and observations. The interview recordings, field notes and reflective notes were transcribed and checked for accuracy.

### Documentary analysis

We sampled documents about the organisations under study produced by external sources (reports by the regulator and national audit provider organisation), and internally produced documents (e.g. organisational quality strategy, clinical audit policy, audit training materials, reports to and minutes from governance committees). Documents were accessed through the hospital research department and/or participants.

### Stakeholder involvement

The co-production group included three carers, three clinical leads for dementia and three organisation clinical audit leads. The advisory group included representatives from the audit co-ordinator (Royal College of Psychiatrists) and the audit commissioner (Healthcare Quality Improvement Partnership) of the national audit of dementia, professional bodies, patients and researchers studying audit and feedback. The advisory group member perspectives were conveyed to the co-production group by MS. MS is a nurse with qualitative research practice and training, and experience of leading quality assurance and improvement in hospital settings. Prior to data collection, MS facilitated four meetings (8 h) with the co-production group to develop a description of their baseline reported understanding of what happens during audit and feedback, what influences its effectiveness and of potential enhancements. The meetings involved facilitated mixed small group work, presentation and whole-group discussion.

### Data analysis

The interviews and data analysis were undertaken by MS, co-coded by TF. Co-coding involved co-indexing and sorting a sample of data (approximately 100 pages) collated as example quotes across the initial data categories and across all methods, independently reviewing the sample dataset for coding and subsequently challenging the description and categories through joint discussion, until consensus was reached. Extensive exemplar quotes for each category and code were further challenged by all authors at a higher level of abstraction, with credibility enriched through further challenge by the co-production group. Transcript data were entered into Nvivo v12 (QSR International) for data management.

Inductive framework analysis [10] involved becoming familiar with the data through transcribing and reading the observation notes, checking and reading the transcribed interview data and reading the documents. MS identified initial codes and categories from each of the first two observations and interviews. MS compared these across data sources and against the diagrams from the first two interviews in order to create an initial analytical framework. The framework contained both higher-level descriptive headings and more thematic, conceptual issues within those categories. The framework category heading, narrative description and exemplar data extracts were presented to the co-production group alongside their initial views. The extent to which a finding was repeated across sites was included in the presentation. The group considered differences and similarities between the findings, their initial views and their emergent understanding, identified challenges to the analysis and proposed further avenues to explore. This process was repeated twice with additional data and updated categories, before adding explicit group consideration of elements of an existing intervention framework [11], a previous systematic review [2] and theory-informed hypotheses [3]. Two further iterations of presentation and review resulted in a later description which was then used to inform the topic guide at the fourth site. Analysis and presentation of the fourth site findings resulted in only minor amendment to the description; this was identified as an indicator of theoretical saturation of the data.

### Ethics

This study was approved by Newcastle University Faculty of Medical Sciences Ethics Committee (application 01266/12984/2017) and was part of a wider project to describe and enhance audit and feedback in dementia care in hospitals, including both the national audit and local ward audits/monitoring. Only data related to the National Audit of Dementia are included here. Data were collected from January 2018 to April 2019.

## Results

We identified ten different stages to the national audit. These were classified as follows: impetus, agreement to take part, preparation of staff, assessment of care, analysis of data, identification of actions, internal feedback, sense-making, wider organisation feedback and making changes. The stages were common across sites; differences between sites in the content of each stage are described below. Whilst described as stages, there was overlap and interaction between them (e.g. the impetus for participation impacted upon different stages of the audit, notably how data were collected and improvement actions agreed; preparation affected the assessment of practice and the selection of actions; internal feedback affected sense-making and making changes). We found that whilst the collection and dissemination of data were organised at a hospital level, sites went through the above stages (e.g. agreement to take part, making changes) at the wider organisation level. As a result, we describe both hospital and organisation-level findings.

### Impetus, agreement to take part and preparation of staff

There were different drivers to take part in the national audit. These included it being perceived as mandatory, to enable comparison, to report on participation externally and to gain internal resources for improvement:

It (national audit) justifies our existence (as a specialist team), I suppose. And it shows that we’re doing the right thing… [then later] I think our consultant is very proactive in terms of dementia care. She uses the audit as a stick – with the chief execs – to try to improve care (interview 14, dementia nurse specialist).

The role of the national audit as a lever for gaining internal improvement resources will be described in more detail as part of the “Internal feedback and sense-making” section.

Nationally, almost all hospitals that meet the eligibility criteria chose to take part in the national audit (documents 15–18, national audit report). We found that the decision-making process about participation involved a member of the organisation clinical governance team identifying a lead clinician who advised on whether the data could be collected (interview 13, clinical audit facilitator). Their recommendation was reported to an organisation-level governance committee which took responsibility for the decision.

Within three organisations, data collection staff described reluctance to collect data (e.g. observation 9, junior doctor training/audit recruitment):

The junior doctor said they did not want to audit the notes but was asked repeatedly by a consultant, stating that the consultant “just kept pushing me to do it” (observation 23, record review).

There was evidence that the reviewers found data extraction of low value (see the “Assessment of care” section) and uninteresting:

You can see why we only do it for a couple of hours, because it’s so soul-destroying (observation 22, record review).

However, we also found two dementia specialists who attended work to collect data on a non-work day. One of whom had earlier described the audit results as being linked to retaining the dementia role they enjoyed (Table 2).

**Table 2:** Exemplar quotes of further influences upon case note review

Dimension	Exemplar quote
Goals	“We are a little bit fearful because, ultimately, if we were shown not to be making a difference, then what does that say about our team? We haven't done our job? Would our roles be in jeopardy because we haven’t made a difference?” … “if I gave it to the tissue viability team, ‘Why the hell should I audit them?’ Can you imagine if we gave them X amount of notes? They probably would rush through it, and the results would be more negative – because they don’t have a vested interest in the results. I do have a vested interest in the results. That means that, ‘Is this accurate?’ I don’t know.” **(Interview 14, dementia nurse specialist)**
Quality of records	“There’s one (question) around, ‘Is there any evidence in the notes that the discharge plan was discussed with the consultant?’ But nobody writes that.” **(Interview 27, dementia nurse specialist)**
Expectations	“There is certainly an element of, when you expect something not to be there, you don’t look as hard. I suppose there is an element of it, maybe subconsciously, for instance, if I know that there is always a discharge letter and I don’t find it immediately, I will delve deep until I find it. If I didn’t find a ‘This is me’ (patient/carer assessment) after looking through the notes at a cursory glance, would I go that extra mile? Maybe not.” **(Interview 14, dementia nurse specialist)**
Interpretations	During observation 22, the participant verbalised different reasons for recording absence of pain assessment, saying out loud that: it was “not done consistently”, “no expressed pain … recorded, but they haven’t used a tool”, “they’ve put zero but he’s drowsy. They haven’t said whether he’s capable of answering or not” **(Observation 22, record review)**. In contrast, a different reviewer was more lenient in their assessment of practice, deciding that a pain assessment had been undertaken based upon a thumbnail size image where it was possible to see a signature, but not the content of the assessment **(Observation 23, record review)**. A further reviewer interpreted that, if nursing observations are there, then the patient must have had a pain assessment **(Observation 17, record review)**.

To prepare actors to gather data, the Royal College of Psychiatrists provided a guidance document, although this may not be used (observations 11–15, 23, 25). At two organisations, we found the people collecting case note data had impromptu discussions about the interpretation of the standards.

### Assessment of care

The national audit requires collation of data from different sources: an organisational checklist, a staff survey, a carer survey and a review of case notes (documents 15–18, national audit report). Case note review data were largely collected by senior nurses (deputy ward manager level to specialist nurse level) although at one organisation it was predominantly doctors (junior doctor to medical consultant) (observations 16–18, 20–24).

During observation of case note reviews (three organisations *n* = 18), the mean time to review a patient’s notes was 25.7 min (range 9 to 52 min). Most case note reviewers recorded their findings on paper forms. The paper forms were subsequently entered into the national audit web portal by deputy ward manager level staff (mean = 11.3 min per record; range 6 to 20 min) (observation 25, 28). There was evidence from one site that time assessing practice was prioritised over clinical care. At this site, those assessing practice were told by the clinical lead to wear normal clothes to prevent re-assignment to the wards (observation 8) and subsequently a request to undertake an assessment on a patient considered ready for discharge was declined in order to gather data (observation 20).

During data entry, approximately half of the data forms needed to be checked with the data collector (observations 25 and 28). This clarification was not observed, which prevents description of any further interpretation of the standards or case notes, but also implies an underestimation of the total time taken to collect and finalise data.

The observations and interviews showed that the case note reviews were influenced by the quality of record keeping, the case note reviewer’s expectations, interpretations and goals (Table 2). Goals included to complete the data collection task quickly (observation 23), to show the need for investment and to present the team or organisation positively (interview 14, dementia nurse specialist).

Case note reviews were also affected by interpretation of the standards and/or the case notes. There were examples where reviewers had developed their own complex set of unwritten criteria that needed to be met to reach the audit standard; others required a much lower level of evidence. It appeared that less complex decision-making was used by those reviewers who had been reluctant to undertake the case note review and/or wanted to complete the task quickly.

### Analysis of data, identification of actions and wider organisation feedback

Analysis of data is undertaken nationally (documents 15–18, national audit reports), approximately 5 months after the delivery of the care assessed. There is a further 9-month gap between national data entry closing and the release of reports to organisations (document 15–18).

At two organisations, local emergent findings were discussed by those assessing practice with the deputy director of nursing prior to the national reports being received (interviews 18, 27). At all four organisations, the national reports were awaited prior to agreeing actions (e.g. interview 31, deputy director of nursing). Study participants reported that the analysis within the report was robust (interview 3, directorate audit lead) but took a long time (interview 13, clinical audit facilitator; interview 27, dementia nurse specialist).

At each organisation, the national report was shared with a small group (approximately two to six) of positional leaders such as the deputy director of nursing or directorate manager, although they may not read it (interview 6, dementia nurse specialist).

The national report contained 66 pages and had a common structure for every hospital (documents 15–18). It included a description of the audit steering group members and the audit method, the mean scores for England and Wales, a summary of local performance including national ranked position, key national findings and a summary of the local hospital performance on these nationally identified priorities. The report then provided detailed performance information, including data and narrative information summarised from carer and staff surveys. Recommendations for commissioners, board members, clinical positional leaders and ward managers, based upon national performance, were included. In addition, hospital-level data are available in an online spreadsheet.

The clinical leads who develop the organisation’s response described difficulty understanding the national report (interview 6, dementia nurse specialist) and being unclear about how to implement improvement (Table 3). Some clinical leads attended a quality improvement workshop run by the Royal College of Psychiatrists approximately 3 months after the report had been received. The workshop included content about what other organisations had done to achieve their results.

**Table 3:** Analysis of data, identification of actions and wider organisation feedback

Dimension	Exemplar quote
Difficulty understanding the report	“Some of it I had to go and ask people. I think I went just by the key recommendations, in the end, to be honest because it summarised it all for me.” **(Interview 6, dementia nurse specialist)**
Initially unclear about how to implement improvement	“Obviously we understand all of the questions and the reason why we're doing it, but the process isn't necessarily that clear…Definitely what has changed (since undertaking the first National Audit) is the thought process in terms of before it even starts about who we need on board, why we need them on board, what we want them to find or do or see or look at.” **(Interview 6, dementia nurse specialist)**
Relative context is considered	“I had a look at these (results from neighbouring organisations) and I did some comparing. It’s not really fair to compare because the resources in the two Trusts (NHS organisations) are totally different.” **(Interview 18, deputy director of nursing)**
Ward-level staff in all participating organisations may not get feedback	“The matrons would get it (committee paper and verbal feedback on national audit) from me at the Matrons’ Forum. Then we would expect the matrons to feed that back down to ground floor level. But I would say that’s the part that doesn’t happen, people on the ward see it. When we start going to introduce new things and when we talk to them about how we’re introducing it, it’s on the back of the audit. …I honestly don’t believe that happens (feedback at ward level). I can’t think of any ward sister, even on our older people’s wards that would not be aware that we do national audits because they get- We’re always on at them about the carers’ one (survey) and the staff one (survey). But in terms of the audit results, I don’t think it goes that far.” **(Interview 27, dementia nurse specialist)**
Staff may get information about actions	“Following lower than national average scores for discharge planning and carer rating for communication on round 3 of the National Audit of Dementia an action plan to remedy these shortfalls had been accepted by the executive team.” **(Extract from directorate newsletter, document 30)**
Feedback may not alter participants’ understanding of performance	“I don’t know. I suppose they will be fed back, but would they change their practice as a result of it? I don’t know. Really, am I going to change my practice as a result of this audit? No, because I know the deficits anyway,” **(Interview 14, dementia nurse specialist)**

The organisation-identified clinical lead (medical consultant or dementia nurse specialist) translated their organisation’s report(s) into a local standardised template, including proposed actions. The deputy director of nursing may also be involved in writing the internal report (interview 27, dementia nurse specialist).

Whilst the national feedback was at hospital level, information and actions in the internal reports were at the organisation level (documents 7–10, internal reports). The internal reports appeared to focus more on national than local performance, and actions rarely addressed relative or absolute low hospital or organisation performance. For example, one organisation with two sites had ten lower quartile results, but eight of these results did not have actions to address performance (document 7, internal reports). Four hospitals (in three organisations) assessed functioning in fewer than 50% of patients but did not have actions to address performance.

At three organisations, the internal reports describe actions targeting all five of the key national priorities, with three of the five addressed by the fourth organisation (document 9, internal report).

Some participants who wrote the internal report described undertaking analysis to compare with other organisations; this was undertaken to identify high-performers to learn from (interview 27, dementia nurse specialist) and to compare performance against other similar organisations (interview 12, deputy director of nursing). Participants described considering organisational context as part of this comparison (interviews 8, 18, deputy directors of nursing).

Despite this, comparative data were included in only two organisation’s reports (documents 9, 10), both comparing themselves against the national average. There were no examples of alternative external comparisons (e.g. comparison against top performers or a peer group) in any internal reports (documents 7–10, internal reports).

Potential reasons for current performance were not reported in three of the four internal reports (documents 7, 8, 10, internal reports). In the site where they were reported (document 9, internal reports), it was not clear how the barriers to performance were identified or how proposed actions were linked to the identified barriers. Who should be doing the target care behaviour (e.g. assess pain) was considered in the development of the action plan, but this information was not included in the internal report or action plan (observation 10).

Proposed actions in the internal reports often reiterated or amended existing actions (documents 11, 12, organisation dementia strategy) and frequently involved changing the health record, training or further audit (documents 7–10, internal reports). The selection of actions was constrained by the perceived sphere of influence of those writing the action plan. Action plans described what would be done and by when (documents 7–10). Most described who would be responsible, some described the outcome sought and one described the possible obstacles to completing the action.

### Internal feedback and sense-making

At each site, possible actions to improve care were discussed with a small group of stakeholders during the development of the internal report, typically the clinical lead, dementia nurse specialist, and deputy director of nursing. The actions were then amended at specialty (e.g. care of older people) level committees (observation 3, dementia steering group) and further refined and agreed at organisation-level governance committees (observation 1, 5, 31, 35). At one organisation (observation 7, clinical governance committee), presentation at the organisation-level committee led to further discussion prior to agreement.

During committee meetings, positional clinical leaders and senior managers considered whether and how to change. They discussed the motivation of the audit provider (Royal College of Psychiatrists) and the validity of the data (interview 19, 30; observation 35). Verbal feedback supplemented the written report by including information about relative performance, typically describing where the hospital was ranked in the top or bottom six of all participating hospitals. As described as part of the impetus to participate in the audit, low relative performance increased commitment to improve:

I don’t know how valuable the benchmarking is, apart from that it brings it to the attention of the board. If you’re somewhere near the bottom then they want something done about it, it’s a useful lever sometimes in that way (interview 18, deputy director of nursing).

However, comparison can also lead to complacency:

So, gather data and then see that a lot of organisations are in a similar position, so it’s accepted that that’s the position that it is… I think sometimes there’s a degree of a) complacency or b) it’s not possible to improve (interview 19, deputy director of nursing).

One participant described absolute performance as more important as a lever for improvement than relative performance:

I’m not so bothered about the difference (between two hospitals), I’m bothered that only 60% of them got one (a discussion about discharge with the carer). What would bother me more, would be why they weren’t being done, because that’s just over half. Two thirds isn’t good (interview 11, directorate manager).

Participants and observations revealed how data may be triangulated with other organisation data. This was a narrative process during meetings (observations 1, 5, 7, 30, 31, 35). Patient experience data, complaints (e.g. observation 8) in particular, were often used as a measure of “true performance”; that is, that national audit data appeared to be viewed as credible if it agreed with issues raised in complaints. Informing this discussion with narrative case studies may support the engagement of influential positional leaders (observation 5, clinical governance committee).

Through this sense-making work, concerns may be added to the hospital risk register and scored. The risk score allocated is affected by external pressure, including from regulatory, reputational and financial risk (interview 20, clinical governance facilitator; document 36). Some seek to game the risk level by changing it to affect who becomes aware:

The clinical audit facilitator said, “all audits on forward plan get risk rated…if less than 12 they are not escalated”. We “try to keep it at the lower end, nine times out of ten they are…if higher they get discussed at Board.” The dementia nurse specialist said that you do not want the Board’s input as it was, “a hindrance not a help” (observation 2, clinical audit facilitation meeting).

### Wider organisation feedback

Ward-level staff in all participating organisations may not get feedback on the national audit results. However, there was evidence that ward staff may get information about actions being taken to improve (Table 3).

Across the sites, dementia specialists described having a good understanding of the anticipated results. As such, feedback may not alter participants’ understanding of performance, and this may affect whether it leads to action (Table 3).

### Making changes

At each organisation, actions were monitored (interviews 1, 13, 20). Monitoring demonstrated that actions were sometimes delayed (documents 24–27, observations 5, 31). Some action owners were unaware of the actions for which they had been assigned responsibility (interview 11, directorate manager) and some action owners leave and the actions are not completed (interview 27, dementia audit lead). Across each organisation, many participants (e.g. interview 14, 24, 27; observation 8, dementia steering group) reported that the next report of the national audit comes too soon (from July 2017 to July 2019) to see improvements in the results.

## Discussion

There is scope to improve returns on investment by enhancing the content and delivery of national audits [6]. In order to identify potential enhancements to the national audit of dementia, we studied how six hospitals completed the audit. Our analysis of documents, interviews and observations, and the co-produced description revealed similar stages to the national audit of dementia across the sites. We found there was an organisation-level decision-making process about participation, where the default position was to take part in national audit, and identification of a lead. The identified lead, typically a dementia nurse specialist or medical consultant, took steps to prepare those involved in data collection. The collection of audit data was affected by record keeping, and case note reviewers’ expectations, interpretations and goals. Nationally, the audit included 10047 case notes; our findings suggest that this equates to almost 6200 h of senior (e.g. deputy ward manager or above) clinical time, with only one identified example of data entry being undertaken by a non-clinician. Importantly, this estimate excludes work to agree to take part, preparation and clarification work, analysis of data or the identification and completion of actions to improve.

The data were analysed nationally; this was reported as being robust but slow. Hospital performance and national performance (mean and hospital ranking) were described in a report sent to the organisation clinical leads. The report was read by few people. The organisation clinical lead for the audit translated the report into an internal report. The leads often found the national report confusing and were uncertain how to create organisational change. The internal report acted as a filter, with subsequent conversations about actions being based only upon the internal report. There was little evidence that actions within the internal report were informed by a robust analysis of current performance involving the identification of low performance, specification of the target care behaviours, analysis of the causes of performance, and the selection of actions to address the causes of performance.

Committee discussion considered relative performance, data quality, narrative triangulation with other data, an assessment of risk and discussion about existing actions. This led to discussion of, and approval for, a plan to improve. The improvement actions may be delayed or not completed.

The study explored what happens within six hospitals as part of four NHS organisations. These were selected for diversity; however, we cannot assume transferability to other audits, institutions or countries. Data from the fourth site sought to test earlier findings. A limitation of the study is that the timing of data collection did not allow for observation of the national audit; however, we were able to explore the content and delivery of the audit through interview and documentary analysis. A further limitation was that the focus was on what happens within the hospital; wider stakeholders (e.g. regulators, commissioners) were not included as contributing participants. Future studies may seek to understand how these wider stakeholders interpret and use the data.

The healthcare workers in the co-production group were from three of the participating study sites. To aid reflexivity, we facilitated the co-production group to describe their pre-study views. MS facilitated the group to challenge emergent findings through explicit consideration of the group’s pre-study views, evidence, theory and an intervention framework. We sought diverse perspectives by involving carers, people from diverse organisations and feedback loops to the advisory group. Sampling was informed by the co-production group members proposing the job titles of key actors in the process, to enable appropriate targeting of participants; sampling was further informed by emergent findings and the advisory group. As a result, whilst it is possible that the involvement of people from the study sites adversely affected the gathering or interpretation of the data, we anticipate that, by drawing upon their knowledge, involvement strengthened the description of the national audit. Concurrent data collection using different methods enabled exploration of themes between data sources and is a further strength.

Comparing the content and delivery of the national audit against evidence [3] and theory [4, 5, 12] identifies potential enhancements to the national audit of dementia. We found that feedback was approximately 14 months after care had been delivered, was discussed at senior level and did not reach clinical staff delivering the care, with the exception of selected positional leaders. The lack of feedback to clinicians mirrors previous findings on the English national blood transfusion audit [7]. Providing more specific, timely feedback may enhance effectiveness [4, 5]. Within the hospitals, this could involve individuals, teams or the organisation getting feedback on the extent to which their care meets the standards measured in the audit from case note reviewers at the point of data collection, or at data entry. The data could be delivered through existing feedback mechanisms (e.g. supervision, ward huddle). Feedback reach is a key implementation and evaluation challenge [13], where actors may receive but not engage with [14], internalise or initiate a response [15]to the feedback. Future research should assess co-interventions to support both delivery of and participation with feedback. Changes to the national reporting process would involve the audit co-ordinator (Royal College of Psychiatrists), the audit commissioner (Healthcare Quality Improvement Partnership) and the audit funder (NHS England) agreeing and delivering more timely and specific feedback reports (e.g. National Hip Fracture Database [16]).

CP-FIT [5] proposes that organisation-level response and senior management support may make feedback interventions more effective. We found that the response to the national audit was at an organisational level. Like Gould et al. [7], we found that the hospital report is received by the organisation clinical lead who leads the organisational response. We found that the clinical leads may be confused by the report. The hospital report from the Royal College of Psychiatrists compares performance against the national mean. Simplifying the report [4, 5] and guiding the tailored selection of comparators [4, 5, 12] may enhance the organisation-level response. The report may be simplified by: reducing the length from 66-pages [9] through the use of interactive reports that enable recipients to get more information if they want it [4, 16], simplifying and standardising graphical presentation [4, 5], highlighting recipient hospital priorities [4, 5] and removing national priorities from the hospital report. These enhancements may require contractual changes from the audit commissioner, such that interactive rather than static (e.g. pdf) reports are produced. Interactive reports could allow recipients to select comparators to highlight room for improvement [4, 5, 12], (e.g. to compare their hospital with the top 10% performing hospitals, or a peer group). Selecting a comparator based upon peer group may increase competition, credibility [12] or fairness (e.g. interview 18).

The organisation clinical leads were unsure how to deliver improvement; many actions described in the internal reports written by the clinical leads did not address local poor performance and were not informed by a robust analysis of current performance. Supporting clinical leads to identify local relative and absolute poor performance [3], to explore influences upon performance and the selection of strategies that address barriers and facilitators [4, 17–19] may enhance improvement as a result of the national audit. This could be done through the provision of interactive data enabling recipients to select absolute and relative performance parameters (e.g. to highlight local quartile results). Facilitation and education [5] could further support recipients to undertake this selection, explore influences upon performance, and select improvement strategies. Providing selectable examples of potential improvement actions for each result, rather than a report-level list of recommendations, may further enhance audit effectiveness [20] by prompting clinical leads to tailor actions to local performance and context.

We found that action plan discussions at speciality- and organisation-level committees included consideration of whether and how to change. This reflects Weiner’s [21] description that commitment to change results from change valence, which may stem from consideration of whether the change is needed, important, beneficial or worthwhile. We found that commitment was related to a discursive assessment of trust in the data, based upon assessment of the source and method, triangulation with recalled data about patient experience, and actors’ assessment of risk, in particular regulatory and reputational risk. This sense-making discussion may generate “legitimacy” [16] and echoes findings from other sources of feedback [22]. Valid and reliable collection of data, and internal organisational feedback that describes the method and triangulated data, may support perceptions of trust and credibility by feedback recipients. Justifying the need for change by linking it to recipient priorities [4, 5] (such as patient outcomes, reputational risks) may further enhance the effectiveness of the national audit.

## Conclusion

We used multiple methods to co-produce a description of the content and delivery of the national audit of dementia at six diverse English hospitals. We found considerable organisational commitment to the audit. We were able to identify potential enhancements to the national audit of dementia that could enhance effectiveness. The enhancements related to the content of the feedback from the national audit provider, support for the clinical leads leading the organisational response to the feedback and the content of the feedback provided within the organisation. We recommend further work to consider whether and how these potential enhancements could be operationalised and the steps needed to implement and test them.

## Figures and Tables

**Fig. 1:**
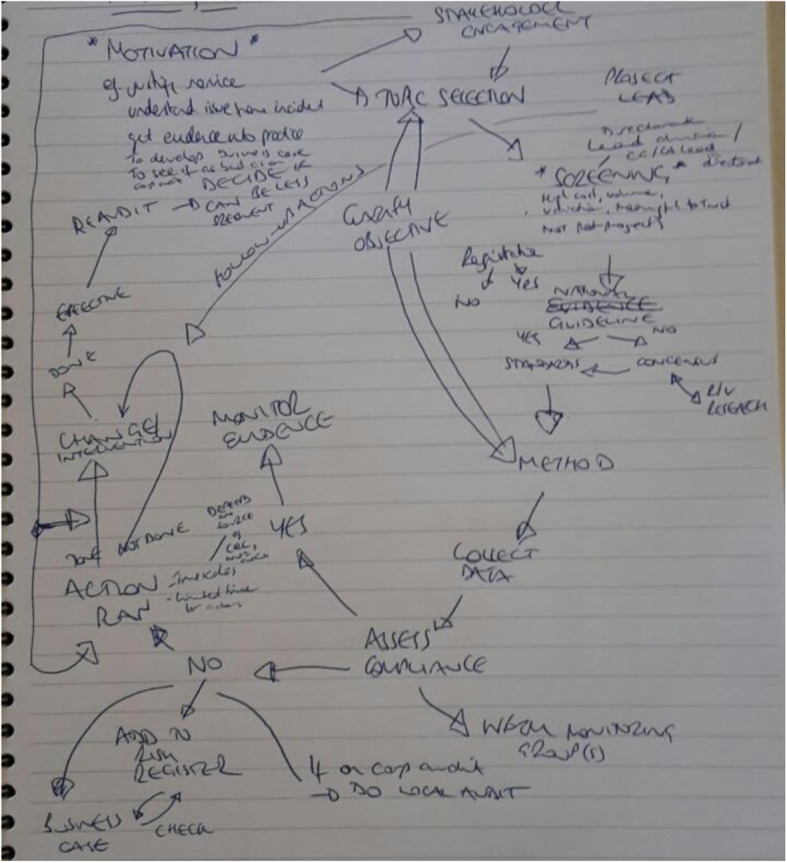
Example interview diagram (interview 1, Organisational Clinical Audit Lead)

**Fig. 2:**
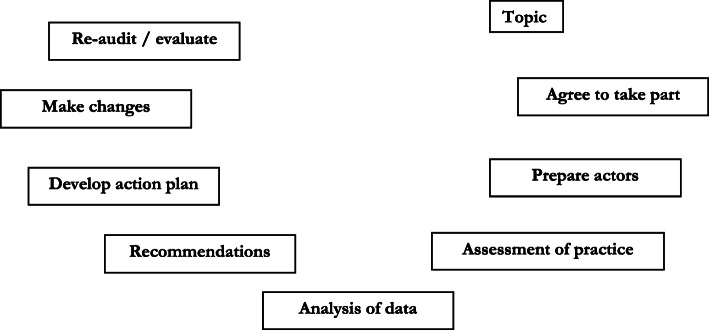
Example collated diagram (version 3) used as interview prompt

## Data Availability

The datasets generated during and/or analysed during the current study are not publicly available in order to maintain the anonymity of participants.

## Appendix 1

**Table 4:** The roles of the interview participants

Role	***n***
Deputy directors of nursing	6
Governance staff	6
Specialist nurses	4
Directorate managers	4
Ward managers	3
Staff nurses	2
Allied health professionals	2
Matrons	2
Medical consultants	2
Executive director of nursing	1

**Table 5:** A description of the observations undertaken

Ref	Title	Description
1	Clinical effectiveness committee	Organisation-level meeting held in board room, including presentation about national audit. 17 attendees, interviewees 1 and 6 present. The committee reports to the clinical governance committee (observation 5).
2	Clinical audit facilitation meeting	Meeting between dementia nurse specialist and clinical audit facilitator to plan the data collection for the national audit.
3	Dementia steering group	Meeting chaired by consultant to discuss improvements in dementia care, includes interviewee 6 and 24.
4	Junior (F1) doctor training and audit recruitment	Meeting to provide training to junior doctors and to seek involvement in data collection.
5	Clinical governance committee	Organisation-level meeting that reports to the Organisation Board. Presentation about national audit. 15 attendees, including interviewees 5 and 6.
6	National audit preparation meeting	Meeting between dementia nurse specialist (interviewee 6), organisation quality assurance lead and clinical audit lead (interviewee 1) to plan the data collection for the national audit.
7	Clinical governance meeting	Organisation-level meeting including presentation about national audit. 36 attendees.
8	Dementia steering group	Meeting chaired by consultant to discuss improvements in dementia care, 10 attendees including interviewee 14.
9	F1 training and audit recruitment	Meeting to provide training to junior doctors and to seek involvement in data collection.
10	Dementia steering group	Meeting chaired by consultant to discuss improvements in dementia care, 9 attendees including interviewee 6 and 24.
11	National audit preparation meeting	Meeting between dementia nurse specialist and ward manager.
12 - 15	National audit preparation meeting	Meeting between dementia nurse specialist and ward manager.
16	Record review	Data collection by dementia nurse specialist.
17	Record review	Data collection by consultant.
18	Record review	Data collection by dementia nurse specialist.
19	Dementia steering group	Meeting chaired by consultant to discuss improvements in dementia care.
20	Record review	Data collection by dementia nurse specialist.
21	Record review	Data collection by dementia nurse specialist.
22	Record review	Data collection by dementia nurse specialist.
23	Record review	Data collection by junior doctor (F2).
24	Record review	Data collection by dementia nurse specialist.
25	Data entry	Data entry by band 6 staff.
26	Ward meeting	Multidisciplinary huddle meeting to discuss both patient care and more general issues.
27	Directorate governance meeting	Specialty quality assurance meeting that reports to organisation-level committee.
28	Data entry	Data entry by band 6 staff.
29	Ward meeting	Multidisciplinary huddle meeting to discuss both patient care and more general issues.
30	Directorate governance meeting	Specialty quality assurance meeting that reports to organisation-level committee.
31	Organisation Clinical Effectiveness meeting	Organisation-level meeting held in Board room, including presentation about national audit. 10 attendees, interviewee 30 present. Committee reports to the Clinical governance committee.
32	Ward meeting	Multidisciplinary huddle meeting to discuss both patient care and more general issues.
33	Ward meeting	Multidisciplinary huddle meeting to discuss both patient care and more general issues.
34	Record review	Data collection by nurse.
35	Clinical governance meeting	Organisation-level meeting that reports to the organisation Board. Presentation about national audit. 11 attendees, including interviewee 18.
36	Clinical audit project meeting	Project meeting to discuss set up of new audit process, 7 attendees including interviewee 19.

## Appendix 3

Interview topic guide v3

1. Could you describe your role?
2. What do you understand by the term audit and feedback? (Can prompt that often called “clinical audit”)
3. Different types of audits have been described.
  - Do you recognise these types? (Show list)
  - Which of these types are you involved with?
  - How do they differ?
4. Do you come into contact with the national audit of dementia? What is your involvement with that audit?
5. The national audit has been described like this (Show collated diagram). Do these match your experience? With which parts of this do you get involved?
6. Explore each part:^1^
  - When does it happen?
  - Where does it happen?
  - Who is involved?
  - How is it done? Is it always done like that? (If appropriate, prompt about materials involved)
  - How does that feel? How do other people feel about that?
  - Which documents and/or potential observations/interview participants could provide more information about this part?
7. Audit is used for different reasons, why do you think it is used here? Any other reasons?
8. Some people use audit to improve care. What do you think about that?
  - How much do you think it improves care?
  - How does it improve care?
  - What affects whether it improves care? (Use collated diagram as prompt)
9. If you could change anything about the national audit, what would you change?
10. What would happen if the hospital didn’t do the national audit? (Can prompt: Would things be different, and if so, how?)
11. Is there anything else you would like to add?

Thank you

## Abbreviations

CP-FIT: Clinical Performance Feedback Intervention Theory
e.g.: Example
NHS: National Health Service
